# Role of the N-terminal transmembrane domain in the endo-lysosomal targeting and function of the human ABCB6 protein

**DOI:** 10.1042/BJ20141085

**Published:** 2015-03-20

**Authors:** Katalin Kiss, Nora Kucsma, Anna Brozik, Gabor E. Tusnady, Ptissam Bergam, Guillaume vanNiel, Gergely Szakacs

**Affiliations:** *Institute of Enzymology, Research Centre for Natural Sciences, Hungarian Academy of Sciences, Budapest 1117, Hungary; †Institut Curie, Centre de Recherche, Paris F-75248, France Structure and Membrane Compartments CNRS UMR144, Paris F-75248, France; ‡Cell and Tissue Imaging Facility, Infrastructures en Biologie Sante et Agronomie, Paris F-75248, France

**Keywords:** adenosine 5′-triphosphatase (ATPase), ATP-binding cassette (ABC) transporter, ATP-binding cassette, subfamily B (ABCB) 6, lysosome, trafficking, transmembrane domain, ABC, ATP-binding cassette, DPBS, Dulbecco’s modified phosphate-buffered saline, HA, human influenza haemagglutinin, HRP, horseradish peroxidase, Lan, Langereis, MDR, multidrug resistance protein, MRP, multidrug resistance-associated protein, NA, numerical aperture, NBD, nucleotide-binding domain, NEM, *N*-ethylmaleimide, NP-40, nonyl phenoxypolyethoxylethanol-40, PAG, Protein A–gold, PFA, paraformaldehyde, P-gp, P-glycoprotein, PNGase-F, peptide *N*-glycosidase F, RT, room temperature, TfR, transferrin receptor, TMD, transmembrane domain, TMD_0_, N-terminal transmembrane domain

## Abstract

ATP-binding cassette, subfamily B (ABCB) 6 is a homodimeric ATP-binding cassette (ABC) transporter present in the plasma membrane and in the intracellular organelles. The intracellular localization of ABCB6 has been a matter of debate, as it has been suggested to reside in the mitochondria and the endo-lysosomal system. Using a variety of imaging modalities, including confocal microscopy and EM, we confirm the endo-lysosomal localization of ABCB6 and show that the protein is internalized from the plasma membrane through endocytosis, to be distributed to multivesicular bodies and lysosomes. In addition to the canonical nucleotide-binding domain (NBD) and transmembrane domain (TMD), ABCB6 contains a unique N-terminal TMD (TMD_0_), which does not show sequence homology to known proteins. We investigated the functional role of these domains through the molecular dissection of ABCB6. We find that the folding, dimerization, membrane insertion and ATP binding/hydrolysis of the core–ABCB6 complex devoid of TMD_0_ are preserved. However, in contrast with the full-length transporter, the core–ABCB6 construct is retained at the plasma membrane and does not appear in Rab5-positive endosomes. TMD_0_ is directly targeted to the lysosomes, without passage to the plasma membrane. Collectively, our results reveal that TMD_0_ represents an independently folding unit, which is dispensable for catalysis, but has a crucial role in the lysosomal targeting of ABCB6.

## Abstract 2

The intracellular localization of ATP-binding cassette, sub family B (ABCB) 6 is a matter of debate. We show that ABCB6 is internalized from the plasma membrane to multivesicular bodies and lysosomes. Molecular dissection of the ABCB6 protein reveals a role of its N-terminal domain in targeting.

## INTRODUCTION

ATP-binding cassette (ABC) transporters are large membrane-spanning multidomain proteins promoting the ATP-dependent transmembrane transport of a vast array of biological compounds, including drugs, bile acids, peptides, steroids, ions and phospholipids [1]. The human genome encodes 48 ABC transporters, which have been grouped into subfamilies labelled A–G. Human ABC transporters are implicated in several diseases, including cystic fibrosis, retinal degeneration, cholesterol and bile transport defects, anaemia and drug response [2,3]. Functionally active ABC transporters contain a minimum of two highly conserved hydrophilic cytosolic nucleotide-binding domains (NBDs) and two hydrophobic transmembrane domains (TMDs), which are encoded by the same gene in the case of ‘full transporters’. In contrast, ‘half-transporters’ possess only a single NBD and a single TMD (together forming the ‘ABC–core’) and must form homodimers or heterodimers to generate a functional ABC transporter. Crystal structures of ABC transporters confirm that the two TMDs provide the substrate-binding sites and form the translocation pore; whereas a dimer of two NBDs bind and hydrolyse ATP [4]. In addition to the ABC–core, some ABC transporters contain further membrane-embedded regions, whose functions are less characterized. Several members of the ATP-binding cassette, subfamily B (ABCB) and ATP-binding cassette, subfamily C (ABCC) subfamilies contain an N-terminal extension to the ABC–core. Long MRPs (multidrug resistance-associated proteins; ABCC1–3, 6, 8 and 10) share a domain arrangement of TMD_0_ (N-terminal TMD)–L_0_–TMD_1_–ABC_1_–L–TMD_2_–ABC_2_, in which TMD_0_ represents an N-terminal five transmembrane helix extension; L_0_ and L represent intracellular linker sequences [5]. Studies of truncated MRP-variants revealed that the intracellular L_0_ (also present in short MRPs) is required for function, but the role of TMD_0_ remains to be clearly established [6,7]. In addition to the well characterized ABCB1 [P-glycoprotein (P-gp)], responsible for the multidrug resistance phenotype of cancer cells [8], the bile salt export pump (ABCB11/BSEP) regulating the excretion of bile salts from the liver and multidrug resistance protein 3 (ABCB4/MDR3) translocating phosphatidylcholine, the ABCB subfamily contains seven half-transporters consisting of a single NBD and a TMD. ABCB half-transporters are expressed in intracellular organelle membranes and must form homo- or hetero-dimers to transport their respective substrates [9]. Interestingly, the N-terminal regions of ABCB half-transporters contain additional, relatively long and unique sequences, which have been implicated in protein–protein interactions and targeting.

The *ABCB6* gene encodes a membrane protein of 842 amino acids, containing a unique N-terminal region followed by the ABC–core consisting of a TMD and an NBD [10]. ABCB6 was first identified as a porphyrin transporter present in the outer membrane of mitochondria [11]. Subsequent studies challenged this conclusion and suggested other localizations, including the plasma membrane [12] and the endo-lysosomal compartment [13,14]. In line with the latter findings, ABCB6 was also found in red blood cells and in exosomes released from maturing reticulocytes [15]. ABCB6 was identified as the molecular basis of the rare blood group system Langereis (Lan) [16]. Given the uncertainty of the intracellular localization, the proposed role of ABCB6 in the direct mitochondrial uptake of haem synthesis intermediates is questionable. Although the initial findings indicated that loss of one *abcb6* allele in embryonic stem (ES) cells impairs porphyrin synthesis, mice derived from these stem cells were phenotypically normal [17]. Whereas Lan-negative individuals (lacking ABCB6) are healthy; mutations in the *ABCB6* gene have been associated with various conditions such as ocular coloboma [18], dominant familial pseudohyperkalaemia [19] and dyschromatosis universalis hereditaria [20].

ABCB6 is presumed to form a homodimer with each subunit containing an N-terminal extension to the canonical ABC–core. This extra N-terminal segment (TMD_0_) is unique to ABCB6 orthologues as it does not show sequence homology to any other protein. Based on extensive sequence alignments and a consensus constrained transmembrane helix prediction algorithm [5], the N-terminal segment is predicted to contain five transmembrane helices. To study the role of the unique TMD_0_ of ABCB6, we compared the catalytic activity and intracellular targeting of the full-length ABCB6 protein and the N-terminally truncated ABCB6–core complex. We find that TMD_0_ is essential and sufficient for lysosomal trafficking, but is dispensable for ATP binding and hydrolysis.

## EXPERIMENTAL

### Materials

Sodium orthovanadate, *N*-ethylmaleimide (NEM), EDTA, EGTA, pheophorbide-A, haemin, prazosin, coproporphyrinogen-III and protoporphyrin-IX were purchased from Sigma–Aldrich. 8-Azido-[α-^32^P]ATP was obtained from Izinta.

### Mutagenesis

cDNA encoding wild-type human ABCB6 (NM_005689) [12] was mutated by PCR to generate a variant bearing a human influenza haemagglutinin (HA) or FLAG tag at its C-terminus. The truncated 206–ABCB6–core variant was generated by PCR. Site-directed mutagenesis was performed to replace the conserved Walker A lysine at position 629 to methionine (K^629^M), as described previously [15].

### Generation of recombinant baculoviruses

The human *ABCB6* cDNA encoding the full-length 842 amino acid protein was cloned into recombinant baculovirus transfer vector for expression in Sf9 insect (*Spodoptera frugiperda*) cells. Sf9 cells were infected and cultured as described in [21]. Individual clones expressing high levels of the human ABCB6 were obtained by end-point dilution and subsequent amplification. The clone producing the highest yield of the ABCB6 protein was selected by immunoblotting.

### Membrane preparation and immunoblotting

Sf9 cell membranes were isolated as described [22]. Three days after virus transfection, the Sf9 cells were harvested, their membranes were isolated and the membrane protein concentrations were determined by the modified Lowry method [23]. Isolated Sf9 membranes were run on 7.5% Laemmli-type SDS gels and the proteins were electroblotted on to PVDF membranes (Bio-Rad). Immunoblotting was performed according to a standard protocol, by using the anti-ABCB6-567 monoclonal antibody [12], in 1:1000 dilutions and an anti-mouse horseradish peroxidase (HRP)-conjugated secondary antibody (1:20000 dilutions, Jackson ImmunoResearch). HRP-dependent luminescence was developed by the enhanced chemiluminescence technique (ECL).

### ATPase activity measurements

Membrane ATPase activity was measured by colorimetric detection of inorganic phosphate liberation as described in [24], with minor modifications. The reaction mixture contained 40 mM MOPS/Tris (pH 7.0), 50 mM KCl, 500 mM EGTA/Tris, 5 mM sodium-azide, 1 mM ouabain and 20 μg of total membrane protein. The reaction was started with the addition of 3.3 mM Mg-ATP. The vanadate-sensitive fraction was determined in the presence of 1 mM sodium-orthovanadate. The indicated drugs (Sigma–Aldrich) were added in DMSO (final concentration 1%). ATP binding and nucleotide occlusion/trapping were measured as described in [25]. In brief, in the ATP-binding assay isolated Sf9 membranes (100 μg) were incubated under non-hydrolytic conditions in a Tris/EGTA buffer (50 mM Tris/KCl, pH 7.0, 0.1 mM EGTA and 2 mM MgCl_2_) for 5 min at 4°C in the presence of 8-azido-[α-^32^P]ATP containing 0.2 MBq of ^32^P isotope, in a final volume of 50 μl. After incubation, the membranes were irradiated with UV light in the presence of the labelled nucleotide for cross-linking. Membranes were washed (centrifuged at 4°C for 20 min at 15000 ***g*** and resuspended) twice in ice-cold Tris/EGTA buffer. The final pellet was resuspended in 20 μl of Tris/EGTA for SDS/PAGE. In the nucleotide-trapping assays isolated Sf9 membranes (100 μg) were incubated under conditions allowing ATP hydrolysis for 5 min at 37°C in the presence of trapping agents (500 μM sodium orthovanadate or AlF_4_) and 5–50 μM of 8-azido-[α-^32^P]ATP, containing 0.2 MBq of ^32^P isotope, in a final volume of 50 μl. The reaction was stopped by the addition of 500 μl of ice-cold Tris/EGTA buffer containing 10 mM unlabelled Mg-ATP and 500 μM vanadate. The membranes were washed (centrifuged at 4°C for 20 min at 15000 ***g*** and resuspended) three times in ice-cold buffer. The final pellet was resuspended in 20 μl of Tris/EGTA and UV-irradiated at 4°C for 10 min using a Vilber–Lourmat T-15 UV lamp (312 nm, 15 W) positioned 5 cm above the samples. The labelled membrane proteins were separated by gel-electrophoresis and electroblotted onto PVDF membranes. Quantitative ^32^P-labelling was determined by a Phospho-Imager (Bio-Rad). The identity of the 8-azido-[α-^32^P]ATP-labelled bands was verified by immunostaining of the same blot.

### Generation of cell lines

Wild-type ABCB6 and its variants were cloned into pBabe retroviral vectors (AddGene plasmid 1764) and stably expressed in K562 and HeLa cells by retroviral transduction. Briefly, the Phoenix-eco packaging cell line was transfected by using the ExGene transfection system (Fermentas). The cell-free viral supernatant was collected at 48 h after transfection and was immediately used to transduce retrovirus producing PG13 cells. The Phoenix-eco cell line [26] was a gift from G. Nolan (Department of Pharmacology, Stanford University, Stanford, CA, U.S.A.); PG13 cells were obtained from the ATCC. For transient transfection, wild-type ABCB6, 206–ABCB6–core and the N-terminal part of the protein were subcloned into a 3× FLAG–CMV-14 vector (ABCB6–FLAG, 206–ABCB6–core–FLAG and TMD_0_–FLAG respectively). To obtain GFP-fusion proteins, wild-type ABCB6, 206–ABCB6–core and the N-terminal part of the protein were subcloned into a pEGFP–N1 vector (AdGene). Cells were transfected according to the manufacturer's instructions (Fugene HD Transfection Reagent, Promega). The vectors containing GFP-tagged Rab5(Q^79^L) variant were kindly provided by M. Vidal (Université Montpellier II, Montpellier, France).

### Antibodies and dyes

The following primary antibodies were used: anti-ABCB6-567 [12], anti-transferrin receptor (TfR) (Invitrogen), anti-HA (Sigma–Aldrich) and anti-FLAG (Sigma–Aldrich). HRP-conjugated secondary antibodies were purchased from Jackson ImmunoResearch Laboratories. Fluorescently labelled secondary antibodies (goat anti-mouse IgGs conjugated with Alexa Fluor 647 A21235, Alexa Fluor 594 A11005 or Alexa Fluor 488 A10667; goat anti-rabbit IgGs conjugated with Alexa Fluor 647 A21244, Alexa Fluor 594 A11012 or Alexa Fluor 488 A11008) were purchased from Invitrogen. LysoTracker and DAPI dyes were from Invitrogen. The OSK43 antibody was a gift from Dr Yoshihiko Tani (Japanese Red Cross Osaka Blood Center, Osaka, Japan). To label cellular organelles, the following dyes were used: LysoTracker Red DND-99 (Invitrogen), DAPI (Sigma–Aldrich). As isotype controls, reagent-grade IgG from mouse and rabbit serum were used (Sigma–Aldrich).

### Glycosidase treatment

ABCB6–HA- and 206–ABCB6–core–HA-overexpressing K562 cell lysates were treated with peptide *N*-glycosidase F (PNGase-F) enzyme (New England BioLabs) to remove N-glycans. Total protein (20 μg) was analysed by SDS/PAGE, ABCB6 was visualized by the anti-ABCB6-567 antibody.

### Cell culture conditions

The K562 cell line (ATCC) was grown in RPMI 1640 medium supplemented with 10% (v/v) FBS (Invitrogen) and with 2 mM glutamine, 100 units/ml penicillin and 100 units/ml streptomycin (Lonza) at 37°C in a humidified air/CO_2_ (19:1) atmosphere. The HeLa cell line (ATCC) was grown in DMEM (Dulbecco's modified Eagle's medium), supplemented with 10% (v/v) FBS (Invitrogen) and with 2 mM glutamine, 100 units/ml penicillin and 100 units/ml streptomycin (Lonza) at 37°C in a humidified air/CO_2_ (19:1) atmosphere. Cell lines were regularly screened with the *Mycoplasma* Detection Kit (Lonza) and the assays were carried out in *Mycoplasma*-negative cells.

### Co-immunoprecipitation

For immunprecipitation, 80 μl of Protein A/G Agarose beads (Pierce) were loaded on a column (Pierce) and coated with anti-FLAG antibody (10 μg) for 2 h at room temperature (RT), then at 4°C overnight. For each sample, 10^6^ transfected cells (72 h after transfection) at 70% confluency were solubilized in 1 ml of freshly made lysis buffer [45 mM Tris, 135 mM NaCl, protease inhibitor cocktail (Roche), 0.5% NP-40 (nonyl phenoxypolyethoxylethanol-40), 1 mM PMSF and 10 mM NEM] for 5 min on ice. Cells were harvested and sonicated for 10 s. Solubilized cells were centrifuged for 10 min at 13000 ***g*** at 4°C. The supernatant was incubated with the precoated beads for 2.5 h at 4°C, then for 30 min at RT. After washing the beads with PBS containing 0.5% NP-40, the bound protein was eluted with 60 μl of Laemmli buffer for 2 h at RT and 2 h at 4°C. Eluted proteins were separated by SDS/PAGE, transferred to PVDF membranes (Bio-Rad) and visualized by immunodetection.

### Confocal microscopy

Cells transfected by GFP-fusion proteins were incubated with LysoTracker for 30 min at 37°C and living cells were analysed by confocal microscopy. Where indicated, chloroquine was added to the cells at 100 nM for 2 h in serum-free medium. Rab5(Q^79^L)–GFP and the FLAG-tagged protein co-transfected cells at 70–80% confluency were gently washed with Dulbecco's modified phosphate-buffered saline (DPBS), fixed with 4% paraformaldehyde (PFA) in DPBS for 10 min and permeabilized in methanol for 90 s at RT. The samples were then blocked for 1 h at RT in DPBS containing 2 mg/ml BSA (Sigma–Aldrich), 1% fish gelatin from cold water fish skin (Sigma–Aldrich), 0.1% Triton X-100 and 5% goat serum (Sigma–Aldrich). After blocking, the cells were incubated for 1 h at RT with the primary antibody diluted in blocking buffer. After washing with DPBS, the cells were incubated for 1 h at RT with the respective Alexa Fluor-conjugated secondary antibody diluted at 1:250 in blocking buffer. Where indicated, DAPI was diluted in DPBS and added to the cells after the incubation with the secondary antibody for 10 min at RT. HeLa cells treated with Dyngo were incubated for 4 h at 37°C in the presence of 30 μM Dyngo-4a (Abcam). After fixation and permeabilization, the cells were labelled with the OSK43 and anti-TfR antibodies. Surface expression of ABCB6 variants expressed in K562 cells was performed as described previously [15]. Samples were studied with an Olympus IX-81/FV500 laser-scanning confocal microscope, using an Olympus PLAPO 606 [1.4 numerical aperture (NA)] oil-immersion objective [27,28].

### FACS analysis

For FACS analysis, K562 cells were labelled by OSK43 anti-ABCB6 antibody with or without permeabilization. Permeablized cells were first fixed by 4% PFA (10 min at RT) and then permeabilized by 0.1% Triton X-100 (10 min at RT). Antibody staining was performed for 45 min at 4°C by using the OSK43 monoclonal antibody (100× dilution, 0.5 μl/tube) specifically recognizing ABCB6 protein, or without primary antibody (negative staining). After washing out the unbound primary antibodies, secondary antibodies corresponding to the IgG type and labelled with Alexa Fluor 488 were added to the cells [Goat F(ab′)2 fragment anti-human IgG (H+L)-Alexa Fluor 488 (Invitrogen)], at 200× dilution, incubated for 45 min at 4°C, washed and resuspended in PBS containing 0.5% BSA. The labelled samples were subjected to FACS; intact cells were gated based on the forward scatter (FSC) and side scatter (SSC) parameters. Intact cells were analysed for antibody staining by a FACS Attune® Acoustic Focusing Cytometer, Blue/Violet (excitation wavelength: 488 nm for Argon ion laser; emission filters: 530/15 nm for Alexa Fluor 488).

### EM

In EM studies, ABCB6 was revealed by the monoclonal antibody (Santa Cruz Biotechnology); the antibody recognizing CD63 was kindly provided by E. Rubinstein (Université Paris-Sud, France) [29]. For ultrathin cryosectioning and immunogold-labelling, cells were fixed with a mixture of 2% PFA and 0.2% glutaraldehyde in 0.1 M phosphate buffer, pH 7.4. Cells were processed for ultracryomicrotomy and single or double immunogold-labelled using PAG10 (Protein A–gold, 10 nm) or PAG15 as described [30]. All samples were analysed using a FEI CM120 electron microscope (FEI Company) and digital acquisitions were made with a numeric camera (Keen View; Soft Imaging System). The definition of the distinct compartments was based on their morphology and by correlation with immunogold-labelling for CD63 as a marker of late endosomes/lysosomes. Multivesicular bodies were defined as compartments delimited by a membrane with numerous internal vesicles. Electron-dense compartments with vesicular or lamellar membranes were classified as mixed lysosomes. Electron-dense compartments with no internal membranes were classified as dense lysosomes.

## RESULTS

### The ABCB6-core shows membrane insertion, dimerization, ATP binding and hydrolysis

**Figure 1:**
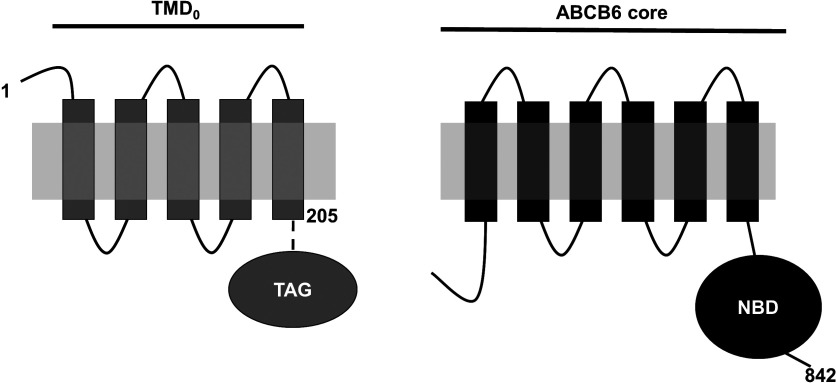
Schematic illustration of the constructs characterized in the present study The core–ABCB6 domain was designed to include the entire intracellular loop preceding the canonical TMD and NBD (residues 205–842). The complementary sequence encoding TMD_0_ contains five predicted transmembrane helices (residues 1–205).

ABCB6 is composed of a core ABC unit (containing a NBD preceded by six transmembrane helices) and an extra N-terminal transmembrane segment predicted to contain five transmembrane helices. Except for the ABCB6 orthologues, the N-terminal segment does not appear in any other proteins. In order to characterize the role of these domains, we created an N-terminally truncated construct lacking TMD_0_. As the cytosolic loop between the extra N-terminal domain and the core–ABC complex was shown to be crucial for functionality in other ABC transporters [7,31], the ABCB6–core was designed to include the entire cytoplasmic loop connecting the fifth and the sixth transmembrane helices (Figure 1).

**Figure 2:**
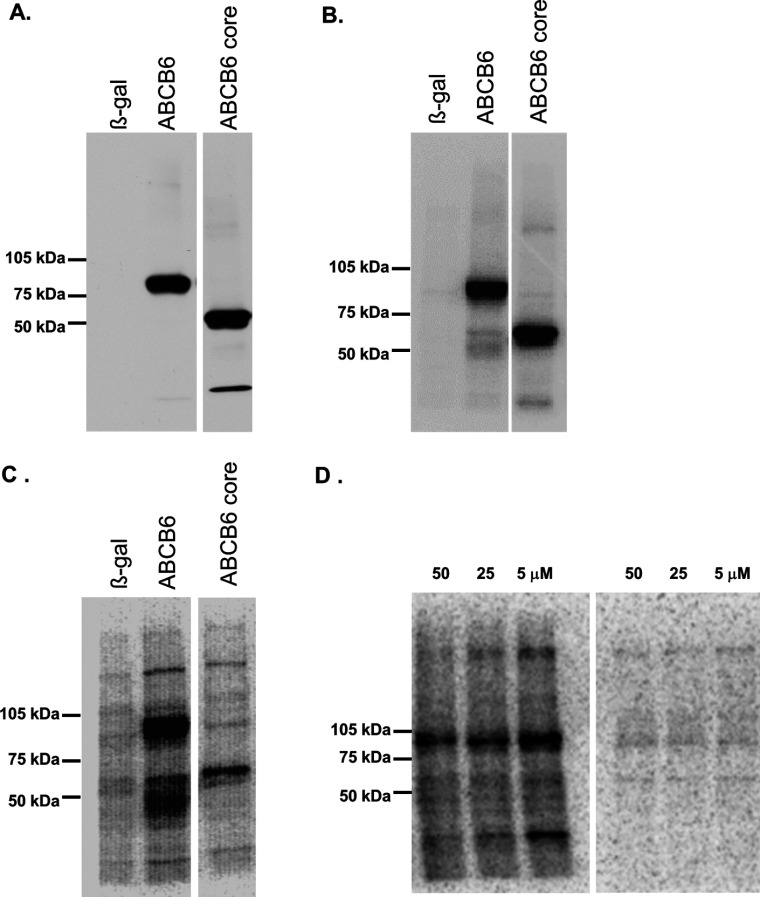
Functional expression of ABCB6 variants in insect cells (**A**) Expression of the ABCB6–core domain in Sf9 insect cells. Isolated Sf9 membranes (2 μg of protein per lane) expressing β-galactosidase (β-gal, lane 1), ABCB6 (lane 2) and ABCB6–core (lane 3) were separated by SDS/PAGE (7.5% gel) and were electroblotted on to PVDF membranes. Immunoblotting was performed using monoclonal anti-ABCB6-567 antibody as described in the Experimental section. Membrane proteins are only core–glycosylated in insect cells [6], which is consistent with the apparent molecular mass of 95 kDa, corresponding to under-glycosylated ABCB6. (**B**) TMD_0_ is not required for ATP binding. Isolated Sf9 membranes expressing β-galactosidase (lane 1), ABCB6 (lane 2) and ABCB6–core (lane 3) were incubated with 5 μM 8-azido-[α-^32^P]ATP under non-hydrolytic conditions (at 4°C) for 5 min, followed by UV irradiation in the presence of the labelled nucleotide as described in the Experimental section. (**C**) TMD_0_ is not required for ATP hydrolysis. Isolated Sf9 membranes expressing β-galactosidase (lane 1), ABCB6 (lane 2) and ABCB6–core (lane 3) were incubated with 5 μM 8-azido-[α-^32^P]ATP and 0.4 mM sodium orthovanadate under catalytic conditions (at 37°C) as described in the Experimental section. Both the full-length and the N-terminally truncated ABCB6–core are capable of ATP binding and hydrolysis. The lower-molecular-mass bands seen in lane 2 correspond to proteolytic fragments and products of vanadate-induced photocleavage [60,61]. (**D**) Mutation of the conserved Walker A lysine is compatible with ATP binding but abolishes nucleotide trapping of ABCB6. Isolated Sf9 membranes expressing-ABCB6-K^629^M were incubated with 5–50 μM 8-azido-[α-^32^P]ATP under non-hydrolytic (left) and hydrolytic (right) conditions as described in the Experimental section.

As shown in Figure 2(A), the full-length and the N-terminally truncated ABCB6–core (206–ABCB6–core) were expressed in insect cells with equal efficiency. Despite our significant experience with measuring the catalytic activity of various ABC transporters [6,22,24,32], the colorimetric assay did not detect any measurable ATPase activity associated with insect cell membranes containing high levels of full-length ABCB6. Thus, we performed additional experiments to verify that the protein is nevertheless capable of binding and hydrolysing ATP [25]. By using Mg-8-azido-[α-^32^P]ATP, covalent photoaffinity-labelling of the ABCB6 variants could be achieved. Experiments performed under non-hydrolytic and hydrolytic conditions proved that both the full-length protein and the N-terminally truncated ABCB6–core are capable of binding and hydrolysing 8-azido-[α-^32^P]ATP, suggesting that loss of the first 205 amino acids does not impede folding, membrane insertion and ATP hydrolysis (Figures 2B and 2C). In contrast, mutation of a crucial Walker A lysine moiety (ABCB6-K^629^M) is compatible with ATP binding, but abolishes nucleotide trapping/hydrolysis (Figure 2D).

**Figure 3:**
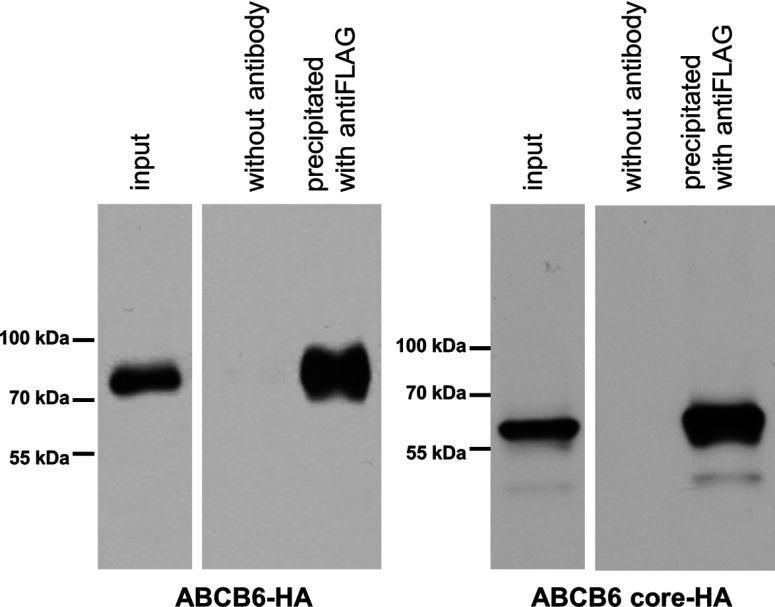
Co-immunoprecipitation of the full-length and the N-terminally truncated ABCB6–core variants Expression plasmids encoding full-length ABCB6 (ABCB6–FLAG and ABCB6–HA), the N-terminally truncated ABCB6 (core–ABCB6–HA) were introduced into HeLa cells. The FLAG-tagged proteins were expressed with the HA-tagged proteins in each set of experiments. The solubilized protein complexes were adsorbed to Protein A/G–agarose beads coated with anti-FLAG antibody. The eluted fractions were separated by SDS/PAGE; the identity of the protein bands was revealed by Western blotting using anti-HA antibodies (see the Experimental section). The ‘input’ lane contains 10 μg of total protein, which corresponds to ~3% of the total sample. The ‘precipitated with anti-FLAG lane’ also contains 10 μg of total protein, which however corresponds to about one-third of the total eluted sample. Thus, there is a significant enrichment of ABCB6 in the immunoprecipitated sample.

ATP hydrolysis requires the co-operation of two NBDs, suggesting that the core–ABCB6 complex can efficiently dimerize. The ability of the ABCB6–core to form dimers was verified by co-immunoprecipitation of HA- and FLAG-tagged ABCB6 variants transiently co-expressed in HeLa cells; the proteins were solubilized from the membranes and analysed by means of a pull-down assay using Protein A/G–agarose beads. In control experiments, the dimerization of the full-length proteins was analysed. In agreement with published data, full-length ABCB6 was found to form homodimers (ABCB6–HA was precipitated with anti-FLAG-coated beads) [11]. Similarly, the N-terminally truncated ABCB6–core formed homodimers, suggesting that TMD_0_ is not required for dimerization (Figure 3).

### Role of the N-terminal segment in intracellular targeting

**Figure 4:**
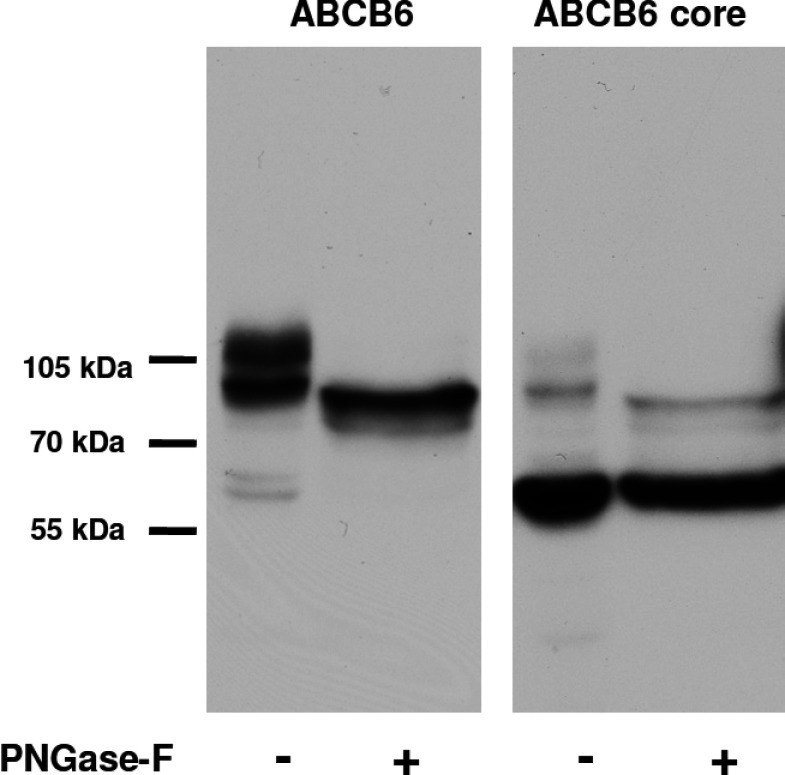
Expression of ABCB6 variants in K562 cells Total lysates of K562 cells transduced with full-length or N-terminally truncated ABCB6 were analysed by SDS/PAGE (7.5%, 20 μg of protein per lane), followed by immunoblotting using the anti-ABCB6-567 antibody. Lysates were treated with PNGase-F enzyme to remove N-glycans. Whereas full-length ABCB6 shows a clear shift after the treatment, there was no change in the case of the core–ABCB6 lacking the N-terminal part of the protein.

The ABCB6–core is sufficient to promote ATP hydrolysis; additional transmembrane helices may be important for the regulation of function or intracellular targeting. To elucidate the role of TMD_0_ in intracellular targeting, we followed the localization of the core–ABCB6 domain. Human HeLa and K562 cell lines were stably transduced by retroviral vectors encoding the full-length or the N-terminally truncated core domain of ABCB6. Expression of the core–ABCB6 domain was confirmed by Western blotting, which showed a single band that could not be shifted by PNGase-F treatment (Figure 4). This is consistent with the glycosylation of ABCB6 on its N-terminal domain, as suggested by a previous study showing that ABCB6 is glycosylated on its N-terminus [33].

**Figure 5:**
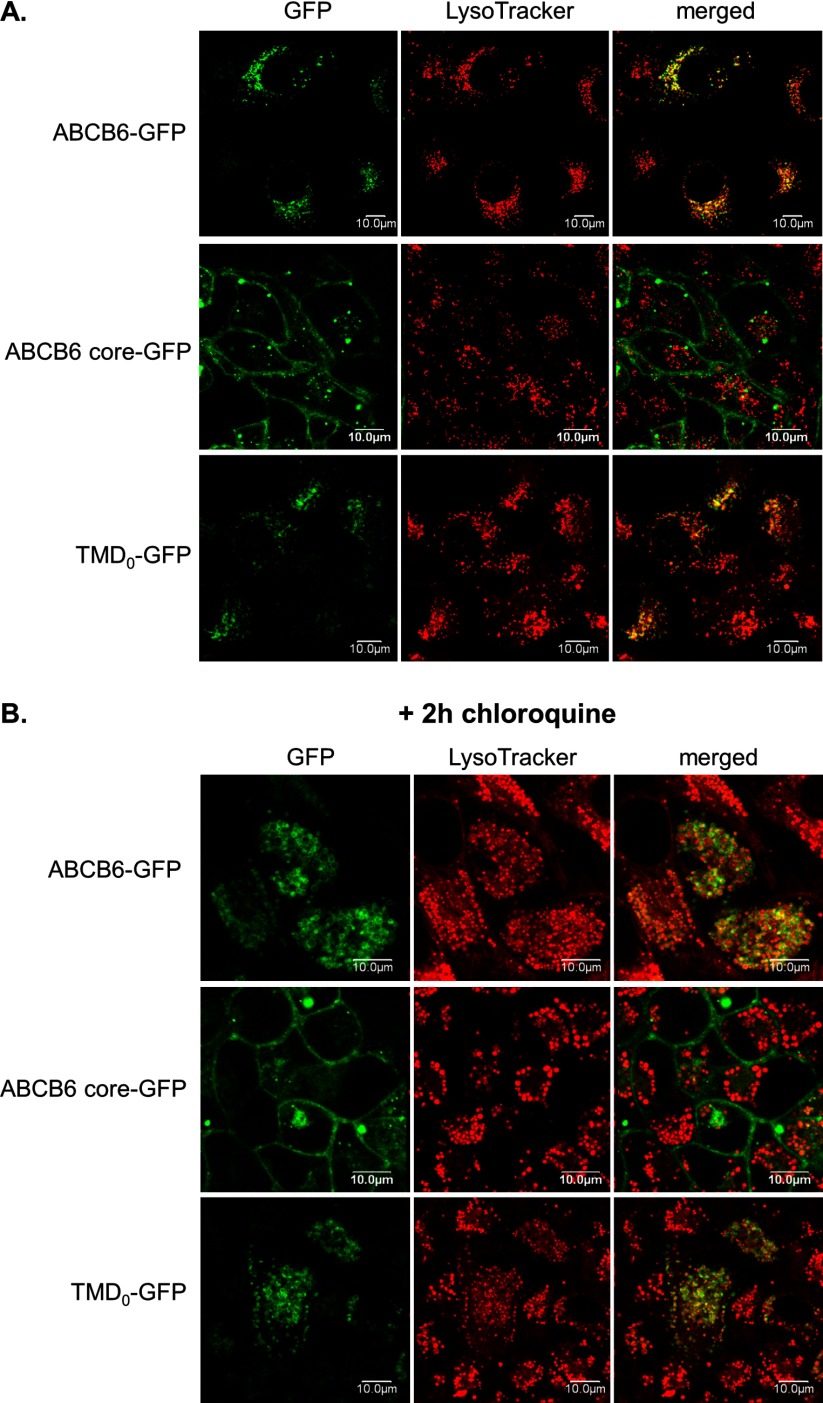
Characterization of the intracellular targeting of GFP-tagged ABCB6 variants in HeLa cells by confocal laser scanning microscopy Cells transfected by GFP-fusion proteins were incubated with LysoTracker for 30 min at 37°C. Localization of ABCB6–GFP, core–ABCB6–GFP and TMD_0_–GFP was revealed by GFP (green) in the context of the lysosomal marker LysoTracker (red). (**B**) Effect of 100 nM chloroquine. Images were collected with an Olympus IX-81/FV500 laser-scanning confocal microscope, using an Olympus PLAPO 606 (1.4 NA) oil-immersion objective [27,28].

We next investigated the subcellular localization of GFP-tagged ABCB6 variants in transiently transfected HeLa cells (Figures 5A and 5B). In accordance with its endo-lysosomal expression, full-length ABCB6 appeared in LysoTracker-positive intracellular compartments. This co-localization persisted after treating the cells with the lysosomotrophic agent chloroquine, which resulted in a characteristic change in lysosome number and morphology. Strikingly, the core–ABCB6 domain showed a distinct localization, appearing dominantly in the plasma membrane. In sharp contrast, TMD_0_ directed the GFP to the endo-lysosomal compartment. As seen with the full-length protein, co-localization of TMD_0_ with LysoTracker was enhanced by chloroquine treatment.

**Figure 6:**
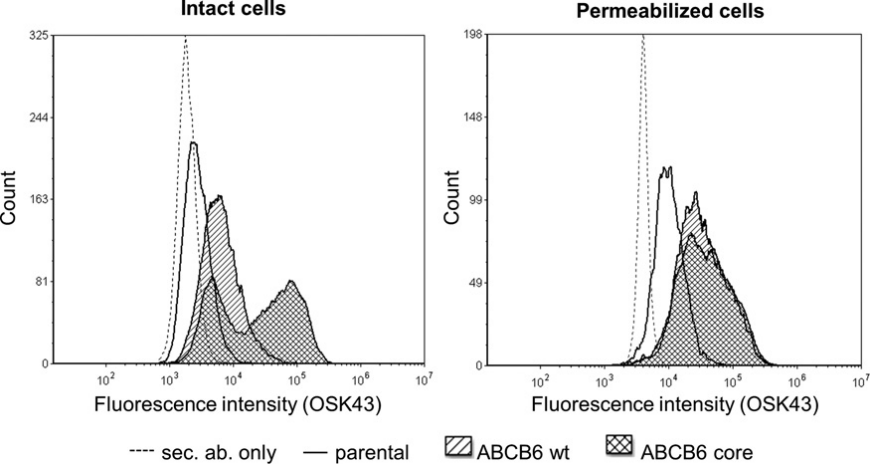
Detection of ABCB6 levels by flow cytometry K562 cells were stably transduced with retroviral constructs encoding ABCB6 or its N-terminally truncated variant (ABCB6–core). Expression of the ABCB6 variants was analysed by flow cytometry using the monoclonal anti-Lan OSK43 antibody. The left and the right panels show histograms corresponding to background fluorescence (secondary antibody only, ‘sec. ab. only’), endogenous ABCB6 expression, the overexpression of the wild-type or the truncated ABCB6 variants, measured in unpermeabilized and permeabilized cells, respectively.

To substantiate the plasma membrane localization of the ABCB6–core domain, we repeated the experiments with K562 cells stably expressing untagged forms of the full-length ABCB6 protein and the core–ABCB6 variant. In the present experimental setup, we benefited from the use of the OSK43 antibody, which recognizes an extracellular epitope of the core–ABCB6 domain [16]. FACS analysis of intact cells demonstrated that the core–ABCB6 domain was predominantly found in the plasma membrane (note that the cells are heterogeneous with respect to the cell-surface expression of the core–ABCB6 domain), whereas the full-length ABCB6 protein could be revealed only upon permeabilizing the cells (Figure 6). These results confirm that ABCB6 is mainly intracellular and the core–ABCB6 domain is predominantly localized in the plasma membrane.

**Figure 7:**
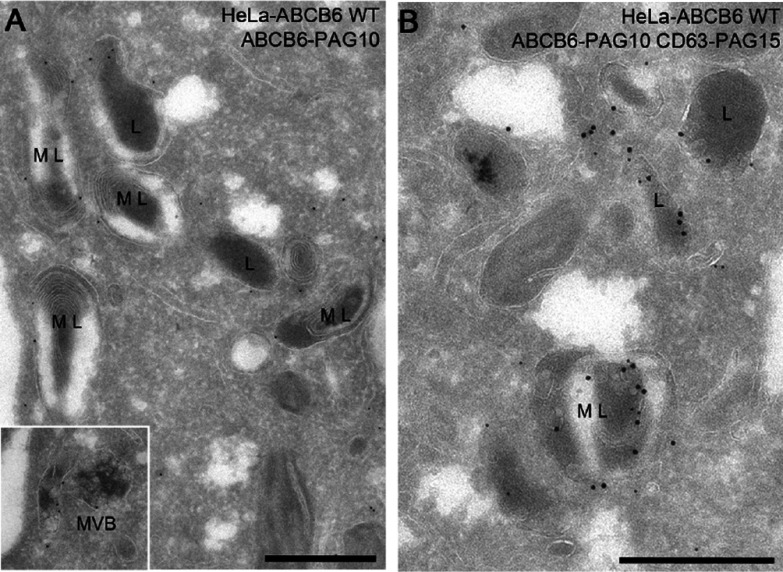
Localization of ABCB6 by EM HeLa cells overexpressing ABCB6 wild-type (WT) were processed for ultrathin cryosectioning and immunogold-labelling. (**A**) Electron micrographs showing the localization of ABCB6 (PAG10). (**B**) Co-localization of ABCB6 (PAG10) with CD63 (PAG15). Multivesicular bodies, multilamellar lysosomes and dense lysosomes are respectively annotated as MVB, ML and L (Scale bar=500 nm).

Finally, the intracellular localization of ABCB6 was analysed by EM. The EM pictures of ultrathin cryosections of HeLa cells overexpressing ABCB6 confirmed the localization of ABCB6 in CD63-positive endosomal compartments including the membranes of multivesicular bodies, multilamellar lysosomes and dense lysosomes (Figure 7).

### ABCB6 is rapidly internalized through dynamin-mediated endocytosis

**Figure 8:**
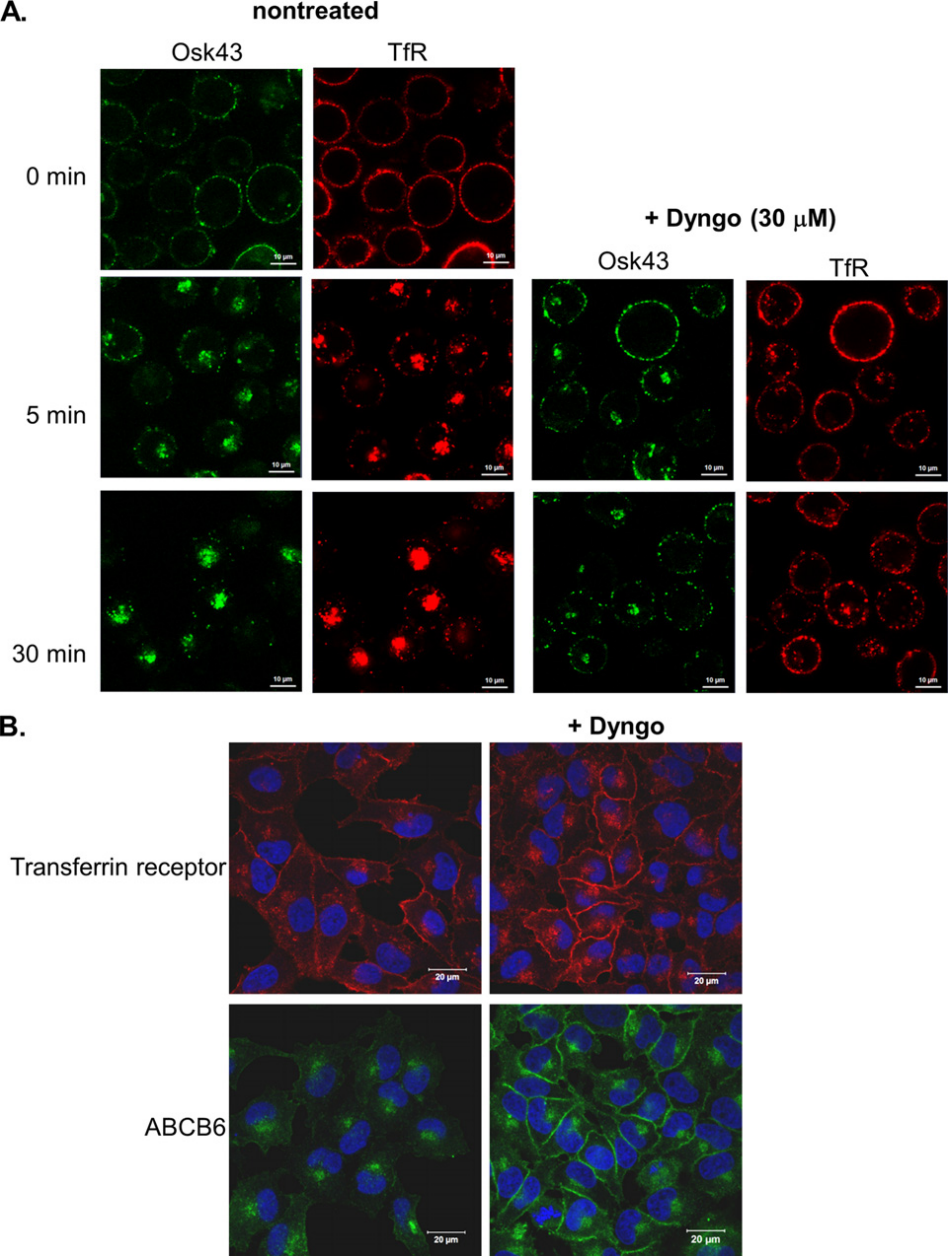
Endogenous ABCB6 is rapidly internalized through dynamin-mediated endocytosis (**A**) K562 cells were pulsed on ice with the OSK43 antibody recognizing an extracellular ABCB6 epitope. Following the removal of unbound antibodies, the cells were placed at 37°C and the OSK43 label (green) was ‘chased’ for the indicated times by confocal microscopy in the presence or absence of 30 μM Dyngo-4a. In control experiments, we followed the redistribution of the TfR (red) that is known to enter the cell through clathrin-mediated endocytosis. (**B**) Treatment of HeLa cells with a highly potent dynamin inhibitor prevents internalization of ABCB6 protein. HeLa cells were incubated for 4 h at 37°C in the presence or absence of 30 μM Dyngo-4a. After fixation and permeabilization the cells were labelled by the anti-ABCB6 OSK43 (green) and anti-TfR (red) antibodies.

Newly synthesized lysosomal proteins can be directly shuttled to the endosomal system from the *trans*-Golgi network. Alternatively, endo-lysosomal localization may be preceded by transit at the plasma membrane compartment. If ABCB6 were to follow the latter route, a relatively rapid internalization of its plasma membrane pool would be expected. To verify this possibility, we followed the redistribution of ABCB6 in K562 cells that express detectable levels of the protein at the plasma membrane [15,34]. Surface labelling of ABCB6 in K562 cells at the initial time point revealed a faint signal corresponding to the plasma-membrane-resident endogenous ABCB6 pool. Similar to the redistribution of TfR molecules, at 37°C, plasma membrane ABCB6 was completely internalized within 30 min (Figure 8A). To investigate the involvement of dynamins and clathrin-dependent coated vesicle formation in the internalization of ABCB6, the pulse–chase experiments were conducted in the presence of Dyngo that acts as a potent inhibitor of endocytic pathways mediating the internalization of plasma membrane proteins [35]. Dyngo treatment significantly reduced the internalization of both ABCB6 and TfR (Figure 8A). Next, we characterized the effect of Dyngo in HeLa cells, which do not express detectable ABCB6 levels on the cell surface. Dyngo treatment blocked the internalization of TfR. Similarly, treatment of cells with Dyngo seemed to completely block the internalization of endogenous ABCB6, as ABCB6 remained on the surface throughout the entire 30-min time course (Figure 8B). Thus, blocking the clathrin-coated dynamin-dependent internalization of endosomes resulted in a marked increase in cell-surface ABCB6 in HeLa cells, suggesting the absence of a plasma membrane pool in these cells is due to rapid internalization.

### In contrast with the full-length ABCB6, the core domain does not appear in Rab5-positive endosomes

**Figure 9:**
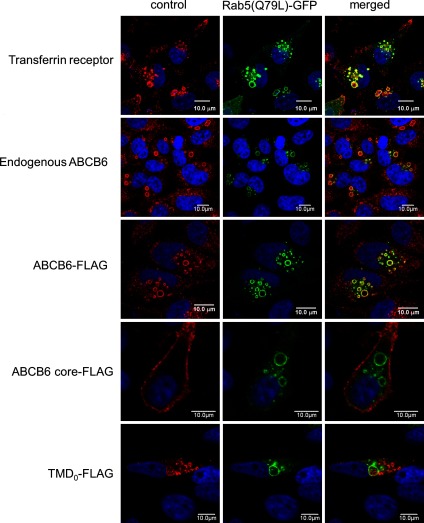
Effect of the constitutively active Rab5 on the distribution of ABCB6 variants within the cell HeLa cells were transiently co-transfected with GFP–Rab5(Q^79^L) (green) and FLAG-tagged ABCB6 variants (full-length, ABCB6–core, TMD_0_; red). Following fixation, the expression of the endogenous TfR and ABCB6 was detected by confocal microscopy, using anti-TfR and anti-ABCB6-567 antibodies respectively, whereas the overexpressed ABCB6 variants were detected by an anti-FLAG antibody (red).

Overexpression of the constitutively active Rab5 mutant [Rab5(Q^79^L)] results in the formation of enlarged cytoplasmic vesicles that exhibit many characteristics of early endosomes including immunoreactivity for Rab5 and TfR [36]. To determine whether internalization of ABCB6 is mediated through early endosomes, ABCB6-expressing cells were transiently transfected with plasmids encoding a GFP-tagged form of Rab5(Q^79^L). As expected, the enlarged vesicles were positive for GFP and the internalized TfR (Figure 9). Similarly, increased Rab5 function resulted in the redistribution of both endogenous and overexpressed ABCB6 into the membranes of GFP-positive intracellular vesicles. In sharp contrast, the N-terminally truncated ABCB6–core labelled the plasma membrane and did not show accumulation in the enlarged vesicles corresponding to early endosomes, suggesting that, with a lack of TMD_0_, the ABCB6–core is not recognized by the endocytic machinery. TMD_0_ did not accumulate in the giant vesicles or the plasma membrane, suggesting that it is directly targeted to the lysosomes, without a detour in the plasma membrane.

## DISCUSSION

The homodimeric ABC transporter ABCB6 was initially identified in mitochondria [11,12] and was later also found in the endo-lysosomal compartment [14,15], the Golgi apparatus [37] and the plasma membrane [12,15,34,38]. Although we cannot exclude mitochondrial localization of ABCB6, in our hands, imaging with a variety of modalities of cells expressing endogenous or overexpressed ABCB6 failed to ascertain targeting to the mitochondria. Our results show that ABCB6 is internalized from the plasma membrane through endocytosis, to be distributed to multivesicular bodies and lysosomes. Using EM we show, for the first time, that ABCB6 is localized in the membranes of multivesicular bodies, multilamellar lysosomes and dense lysosomes.

Our major aim was to delineate the role of ABCB6's unique N-terminal domain in regulating ABCB6 function and intracellular targeting. The N-terminal extension of ABCB6 does not contain recognizable motifs or homology to other proteins. As the cytosolic loop between the N-terminal TMD_0_ and the core transporter is crucial for functionality in some ABC transporters, we assigned the domain boundary between the core–ABCB6 and TMD_0_ to residue 205, which is immediately proximal to the predicted five transmembrane helix bundle of TMD_0_. The ABCB6 fragments were expressed both in Sf9 insect cells and in mammalian cells. In the first system, due to the high levels of expression in a heterologous cell type, functional studies and convenient biochemical assays could be carried out on isolated membranes with less interference from other proteins, whereas the mammalian system offered the possibility to efficiently study membrane routing and intracellular trafficking.

Despite high expression levels, ABCB6 did not show a measurable ATPase activity in Sf9 membranes, as may have been expected based on the successful functional expression of several ABC transporters in insect cells, including ABCB1 (MDR1/P-gp), ABCC1–2 (MRP1, MRP2), ABCG1 or ABCG2, that have been extensively characterized in our laboratory [6,7,21,24,25,39–41]. It may be argued that the lack of measurable ABCB6 ATPase activity is due to a defect in the insertion or folding in the insect cell membrane. The high-level expression of a core-glycosylated ABCB6 with a correct molecular mass, the specific Mg^2+^-dependent ATP binding and the formation of a catalytic intermediate strongly argue against this possibility. It is also possible that the ATPase turnover of the ABCB6 is too low to allow a direct detection of ABCB6-mediated ATP hydrolysis against the background of the insect ATPases present in isolated membrane preparations. However, a recent study demonstrated that the purified and reconstituted ABCB6 protein possesses a significant substrate-stimulated ATPase activity [42]. Therefore we measured the vanadate-sensitive ATPase activity in the presence of various compounds suggested to be transported by ABCB6, but found no stimulation when pheophorbide-A (5–100 μM), haemin (5–100 μM), prazozin (5–100 μM), coproporphyrinogen-III (5–50 μM) or protoporphyrin-IX (5–100 μM) were added to the isolated membranes containing ABCB6 (results not shown).

In the presence of ATP, a divalent cation and a transition state analogue, such as vanadate, a nucleotide is trapped by ABC proteins, reflecting a drug-stimulated partial reaction of the ATP-hydrolytic cycle [9,43]. Detailed analysis of the optimum conditions for 8-azido-[α-^32^P]ATP trapping indicated that the formation of the stable (‘trapped’) nucleotide–ABCB6 complex requires incubation at 37°C and the presence of phosphate-mimicking transition state analogues, suggesting that the catalytic turnover does not allow the detection of natural nucleotide occlusion in the absence of inhibitory anions. Mutation of the conserved Walker A methionine residue impairs hydrolysis of several ABC transporters [21]. In the present study, we show that the K^629^M mutation allows ATP binding, but prevents ATP hydrolysis, in agreement with the abolished ATPase activity of ABCB6-K^629^A [42]. In contrast, the core–ABCB6 construct can hydrolyse ATP (Figure 2C). Quantification of the experiments revealed that the core–ABCB6 form shows reduced trapping activity (50%). This result may suggest a drop in the affinity for ATP, a change in the catalytic cycle resulting in a decrease in the average time during which vanadate can replace the terminal phosphate, or an altered conformation in which the labelling efficiency of the azido group is decreased. Labelling was only observed in the presence of vanadate, indicating that the trapped nucleotide species is ADP. Thus, ABCB6 is able to hydrolyse ATP without its N-terminal domain. Since the ATPase activity of ABCB6 seems to be inhibited by a component of the insect cell membrane, a more detailed characterization of the influence of TMD_0_ on the kinetic parameters of ATP hydrolysis will require purified and reconstituted proteins.

Similar results have been obtained with a variety of ‘long’ ABC transporters possessing an N-terminal extension. TMD_0_ was shown to be dispensable for the ATPase and/or transport activity of ABCC1 (MRP1) [6,7] and ABCC2 (MRP2) [44]. Intriguingly, all of the ABCB half-transporters are expressed in intracellular organelles and all have N-terminal regions containing relatively long sequences [5]. Correct folding and preserved function of N-terminally truncated core–ABCB transporters were reported for ABCB9 [31] and ABCB2–3 [45]. It has to be noted that the functionality of the truncated ABC transporters is dependent on the domain boundaries of the constructs. The selection of appropriate boundaries can be difficult unless the structure is already known, since the deletion of critical amino acids may disturb the structure or folding of the neighbouring membrane-associated regions, which may influence function and/or trafficking [7,46]. Although the exact topology of ABCB6 is unknown, the domain boundary of the ABCB6–core was defined to include the entire cytoplasmic loop connecting the fifth and the sixth transmembrane helices.

Although the ATPase activity of the core domains of long ABC transporters was generally preserved, removal of TMD_0_ had variable consequences on trafficking. Core–MRP1 still traffics to the basolateral membrane in polarized epithelial cells [7] and the core–TAP (transporter associated with antigen processing) complex seems to be correctly retained in the endoplasmic reticulum [45,47,48]. In contrast, core–MRP2 is localized in intracellular compartments and is no longer directed to the apical membrane [6]. The core–ABC domain of Ycf1p (yeast cadmium factor 1 protein), the prototypical member of the ABCC subfamily of *Saccharomyces cerevisiae*, fails to reach the vacuoles and accumulates in intracellular compartments [49]. Remarkably, the N-terminally truncated ABCB9 behaves much like the ABCB6–core as it is also targeted preferentially to the plasma membrane [31]. Similar to the results obtained with the TMD_0_ of ABCB9 (containing only four transmembrane helices), the ABCB6 TMD_0_ was able to direct GFP to the endo-lysosomal compartment of HeLa cells, suggesting that it also forms a folding domain containing sufficient information for lysosomal targeting [31].

Newly synthesized lysosomal proteins can be directly shuttled to the endosomal system from the *trans*-Golgi network. The best understood direct pathway is the mannose 6-phosphate receptor (M6PR)-mediated transport of lysosomal hydrolytic enzymes [50]. Alternatively, lysosomal membrane proteins are first targeted to the plasma membrane for subsequent endocytosis. Previously, we have shown that at high expression levels, ABCB6 accumulates in the plasma membrane, whereas under the same experimental conditions the canonical mitochondrial ABC transporters ABCB7, 8 and 10 remained confined to the mitochondria [15]. In the present study, we have followed the intracellular trafficking of ABCB6 in K562 cells that express low, but detectable, levels of the protein in the plasma membrane. Our results indicate that ABCB6 is indeed rapidly internalized from the plasma membrane through the clathrin–dynamin-dependent pathway. Blocking dynamin-dependent internalization resulted in a marked increase in cell surface ABCB6 expression in HeLa cells, suggesting that the absence of plasma membrane pool in these cells is due to a rapid internalization of ABCB6.

Internalized proteins traffic through the dynamic and adaptable continuum of the endo-lysosomal system to be recycled to the plasma membrane or sorted to the lysosomes. The small GTPase Rab5 controls the fusogenic properties of early endosomes through GTP-dependent recruitment and activation of effector proteins. Expression of a GTPase-defective mutant, Rab5(Q^79^L), is known to cause formation of enlarged early endosomes and later endocytic profiles [36]. Membrane components of early endosomes undergo stringent selection so that only a specific cohort is passed on the late endosomes and eventually to lysosomes, where longer-lived proteins escaping degradation contribute to the maintenance and generation of lysosomes [51]. The localization of ABCB6 in Rab5-positive vesicles confirms the presence of ABCB6 in early endosomes and the EM images convincingly demonstrate the localization of ABCB6 in late endosomes or multivesicular bodies and multilamellar lysosomes. These results suggest that ABCB6 is first targeted to the plasma membrane to be internalized by endocytosis.

Notably, the ABCB6–core was not endocytosed, suggesting a role for TMD_0_ in cargo recognition during clathrin-mediated endocytosis [52]. Lysosomal membrane protein trafficking encompasses a complex network of sorting signals and cytosolic recognition proteins [53]. Sorting signals are typically located in cytosolic loops proximal to transmembrane helices. Most signals consist of short linear sequences of amino acid residues including tyrosine-based motifs (NPXY or YXXØ, Ø being a bulky hydrophobic residue) or di-leucine motifs (D/EXXXLL or DXXLL) [53]. Unconventional di-leucine motifs missing the upstream acidic residue and split targeting motifs were also found to be sufficient for lysosomal localization [54,55]. Finally, ubiquitination of cytosolic lysine residues may serve as a signal for sorting at various stages of the endosomal–lysosomal system [56]. The predicted intracellular loops of TMD_0_ do not contain conventional lysosomal sorting motifs. Further studies will address the role of TMD_0_ in trafficking to identify cryptic routing signals or protein interactions responsible for lysosomal targeting [57].

In summary, molecular dissection of ABCB6 revealed that the TMD_0_ and the ABC–core represent independently folding units, responsible for the lysosomal targeting and the ATPase activity of the full-length protein, respectively. Based on the trapping experiments, the ability of the ABCB6–core to hydrolyse ATP seems to be preserved; further work is needed to establish whether TMD_0_ influences the ATPase or the transport function of ABCB6. The physiological function of ABCB6 in the endo-lysosomal continuum remains to be clarified. Although the trafficking pattern of ABCB6 revealed in the present study may vary in a cell-type-specific manner, our results suggest a function for ABCB6 in lysosomes and related organelles such as melanosomes [20,58,59]. Additional work, currently ongoing in our laboratory will be necessary to address this hypothesis.
